# Coal tar creosote abuse by vapour inhalation presenting with renal impairment and neurotoxicity: a case report

**DOI:** 10.1186/1752-1947-1-102

**Published:** 2007-09-24

**Authors:** Thomas F Hiemstra, Christopher OC Bellamy, Jeremy H Hughes

**Affiliations:** 1 Addenbrookes Hospital, Cambridge University Hospitals NHS Foundation Trust, Hills Road, Cambridge, CB2 2QQ, UK; 2 MRC Centre for Inflammation Research, The Queen's Medical Research Institute, 47 Little France Crescent, Edinburgh, EH16 4TJ, UK

## Abstract

A 56 year old aromatherapist presented with advanced renal failure following chronic coal tar creosote vapour inhalation, and a chronic tubulo-interstitial nephritis was identified on renal biopsy. Following dialysis dependence occult inhalation continued, resulting in seizures, ataxia, cognitive impairment and marked generalised cerebral atrophy. We describe for the first time a case of creosote abuse by chronic vapour inhalation, resulting in significant morbidity. Use of the polycyclic aromatic hydrocarbon-containing wood preservative coal tar creosote is restricted by many countries due to concerns over environmental contamination and carcinogenicity. This case demonstrates additional toxicities not previously reported with coal tar creosote, and emphasizes the health risks of polycyclic aromatic hydrocarbon exposure.

## Background

Coal Tar Creosotes are distillation products of coal tar widely used for preserving wood. Creosotes pose a health risk due to carcinogenicity, and are banned from commercial use in many countries. The European Union banned the commercial sale of creosotes in 2003. Creosote volatiles are complex, consisting of almost 300 different compounds [1]. However, creosote abuse by vapour inhalation has never been described.

## Case Report

A 56 year old aromatherapist presented with advanced renal impairment (Blood Urea 26.1 mmol/l, Creatinine 704 μmol/l). She had been fatigued with general malaise for the preceding three months, but denied any constitutional symptoms. She had developed modest nocturia, but had no other lower urinary tract symptoms. She had been previously healthy, and denied the use of any medications or herbal remedies.

Physical examination revealed pallor, hypertension (Supine BP 210/110) and euvolumia, no mucocutaneous lesions, and no uveitis. Urine analysis was positive for protein on stick testing, with negative microscopy. Full Blood Count at presentation confirmed normocytic normochromic anaemia, without eosinophilia. A renal tract ultrasound was performed, and revealed normal sized unobstructed kidneys.

**Figure 1:**
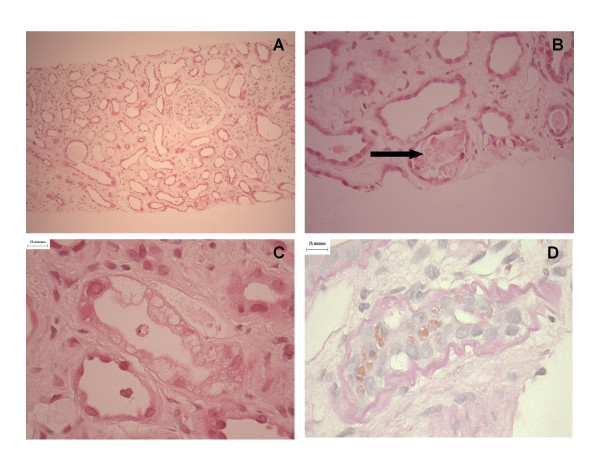
Renal biopsy specimen obtained at the time of clinical presentation with advanced renal impairment. There is severe tubular atrophy and interstitial changes consistent with an advanced chronic interstitial nephropathy. A) Low power haematoxylin and eosin stain demonstrating chronic interstitial nephropathy. B) High power view indicating tubular debris within the nephron lumen (arrowed). C) High power view demonstrating marked tubular cell vacuolisation. D) PAS stain demonstrating thickened basement membrane and marked cytoplasmic lypochrome pigmentation.

Renal biopsy showed a 9 mm core of renal cortex in which the glomeruli and vasculature did not exhibit any significant abnormality. By contrast there was diffuse and striking cortical tubular atrophy with evidence of ongoing epithelial shedding (Fig. 1). The tubular epithelium was markedly attenuated to the point where proximal and distal tubules were not reliably distinguishable. Tubular cells showed occasional apoptosis, increased nuclear variability, scattered tubules showed cytoplasmic vacuolisation, and occasional cells showed cytoplasmic lipochrome pigment. Tubular basement membranes were thickened and wrinkled. The cortical tubules were widely separated by an expanded, rather pauci-cellular interstitial matrix in which sparse small lymphocytes, macrophages and occasional eosinophils were evident. No kidney was present in the immunofluorescence sample, but electron microscopy showed a glomerulus with only mildly expanded mesangial matrix, mild wrinkling of the basement membrane, no immune complex deposits or podocyte foot process fusion. No immune deposits were evident in the interstitium on electron microscopy. It was concluded that the biopsy showed a chronic low grade interstitial nephropathy that was suggestive of a toxin-induced process. Despite re-interrogation of the patient, exposure to all known nephrotoxins was denied. The patient received a trial of oral steroid therapy but failed to improve and commenced regular haemodialysis four months later.

Fifteen months after commencing haemodialysis, the patient was readmitted to the emergency unit with increasing confusion. Admission was followed with three short generalized seizures terminated by intravenous diazepam, with a prolonged depressed level of conciousness post-ictally. As metabolic causes for seizures had been excluded, a Computerised Tomogram (CT) of the head was performed. This did not identify a cause for seizures, but revealed significant cerebral atrophy deemed out of keeping for the patient's age. Magnetic Resonance Imaging (MRI) confirmed cerebral atrophy, and additionally identified subtle deep white matter changes.

Following treatment with intravenous phenytoin she suffered no further seizures though she remained comatose for three days, recovering slowly thereafter. At this time, examination revealed proximal muscle weakness, ataxia, a wide-based gait, blunted affect, and evidence of memory impairment.

A period of in-hospital rehabilitation was followed by a home visit with an occupational therapist, at which time the patient was witnessed to inhale creosote vapour from a container filled with coal tar creosote and kept on a kitchen shelf for this sole purpose. Upon interrogation she admitted inhaling creosote vapour daily and often for at least 6 years prior to the first hospital admission, frequently carrying a concealed supply on her person when venturing from home. Indeed, a neighbour had surreptitiously provided her with a creosote-impregnated cloth in a sealed sandwich container, facilitating continued unidentified inhalation during her inpatient stay.

Further progression of her neurological condition was evidenced by the subsequent development of dementia over a period of months.

## Discussion

Inhalant substance abuse is widespread, typically involving the inhalation of glue, spray paint, petroleum, correction fluid, nail polish remover or dry cleaning fluid by adolescents. Polycyclic Aromatic Hydrocarbons (PAHs) are common to these substances, and their inhalation has multiple toxicities. As all these compounds contain a variety of different PAHs, it is impossible to determine the individual toxicity of any particular PAH. Coal Tar Creosotes likewise contain many PAHs, and creosote vapour includes naphthalene, acenaphthene, fluorene, phenanthrene, anthracene, fluoranthene and pyrene among many others. All of these are detectable in urine following inhalation exposure, with naphthalene being the most abundant [2]. Here we describe the first case of creosote misuse by vapour inhalation.

### Nephrotoxicity

PAH exposure is associated with a higher risk of renal dysfunction and renal cancer. A large body of evidence confirms nephrotoxicity with chronic exposure to PAHs [3], and experimental data have demonstrated both glomerular and tubular toxicity [4].

Specific data on the renal effects of creosote vapour inhalation are limited, although animal studies suggest renal toxicity. Systemic exposure by inhalation has been demonstrated by detection of the pyrene metabolite 1-hydroxypyrene in urine of exposed subjects [5]. In an experimental model of coal tar creosote (CTC) vapour exposure, Springer *et al *observed pigmentation of tubular epithelial cells, proliferation of urothelium, and a significant increase in BUN concentrations [6]. In a cross-sectional study by the Tabershaw Occupational Medicine Associates (TOMA 1981), wood factory workers exposed to creosote had an increased incidence of haematuria, red cell- and granular casts, although renal function of this cohort was not documented. Interestingly this group also noted eosinophilia in 8% of workers.

The smallest and most volatile component in creosote is naphthalene, the principal ingredient of moth balls. Addiction to naphthalene inhalation has been reported, and naphthalene exposure has been associated with chronic renal failure [7].

Mechanistically, a number of factors may contribute to the nephrotoxicity observed with hydrocarbon exposure, as reviewed by Ravnskov [3]. First, hydrocarbons may induce renal injury by combining with renal proteins and acting as haptens to induce auto-immunity against renal cells. Second, hydrocarbons may modify T-cell function leading to an unfavourable cytokine profile and predisposing to T-cell mediated renal injury. This hypothesis is supported by the abrogation of renal injury in animal models by glucocorticoid pretreatment. Third, animal studies demonstrating an effect of species and sex on susceptibility implicate the importance of genetic and hormonal susceptibility.

### Neurotoxicity

Volatile hydrocarbons are rapidly taken up into the circulation, and are sequestered in adipose tissues. Uptake by neurons is particularly rapid, accounting for the rapid 'high' often experienced with inhalant abuse. Subsequent excretion of PAHs or their metabolites occurs via urine, bile and feces. Inhalation of substances containing PAHs have been associated with confusion, disorientation, nystagmus, ataxia, cerebellar degeneration, tremor, white matter degeneration, memory loss, dementia and seizures [8], and there is in vitro evidence for a direct neurotoxic effect of many PAHs present in creosote vapour [9].

However, direct neurotoxicity from creosote vapour inhalation has to our knowledge not been described before. It is important to note that our patient was exposed to creosote vapour for many years prior to presentation, and neurotoxicity prior to dialysis dependence had not been overt. Reports of patients with end stage renal failure due to chronic PAH inhalation do not give the total exposure levels, and so its effects in this population are unknown.

As our patient had become oligo-anuric over the initial 18 months of dialysis dependence, we hypothesize a susceptibility to neurotoxicity in this individual due to a decreased renal excretion of PAHs and their metabolites. The neurological signs were similar to that described with inhalant abuse of other aromatic hydrocarbons like toluene [10], including ataxia, gait disturbance, seizures, generalized cerebral atrophy and deep white matter changes.

## Conclusion

Taken together, the clinical findings of this case and current understanding of PAH toxicities strongly implicate creosote vapour inhalation as the cause of this woman's clinical presentation. The ultrasound findings of 'normal sized kidneys' is perhaps unusual for a chronic interstitial nephritis. It is the case, however, that ultrasonographic estimation of renal size by ultrasound may vary by ~1.6 cm between operators or scans [11] and the histological picture indicates chronic kidney damage. Although causality remains unproven and most histological features are common to any toxin-induced interstitial nephritis, the cytoplasmic pigmentation of renal tubular cells identified on renal biopsy is unusual and congruent with experimental data [6].

As is often the case with inhalant abuse, this diagnosis proved difficult to establish despite repeatedly questioning the patient. Study of urinary hydrocarbons or tubular protein excretion was not undertaken as vapour inhalation was not noted until the patient was dialysis dependent and anuric. Nevertheless our contention that the creosote inhalation was instrumental to the pathogenesis of chronic renal failure is supported by an increasing body of evidence implicating PAH exposure in the development and progression of renal disease. This notion has implications for the significance of occupational exposure to PAHs. Our findings imply an occupational health merit in monitoring renal function in workers chronically exposed to coal tar and its volatiles, a requirement further supported by the possible increased risk of extra-renal toxicity in subjects with pre-existing renal impairment.

Finally, this case highlights the often-underestimated value of assessing patients in the home environment.

## Competing interests

The author(s) declare that they have no competing interests.

## Authors' contributions

TH was responsible for the care of the patient during admissions, and drafted the manuscript. JH was responsible for the patient's management, contributed to the intellectual content, and proof read the manuscript. CB reviewed the renal biopsy samples, contributed intellectual content and photograph, and proof read the manuscript.
